# Modeling Sustained Transmission of *Wolbachia* among *Anopheles* Mosquitoes: Implications for Malaria Control in Haiti

**DOI:** 10.3390/tropicalmed8030162

**Published:** 2023-03-09

**Authors:** Daniela Florez, Alyssa J. Young, Kerlly J. Bernabé, James M. Hyman, Zhuolin Qu

**Affiliations:** 1 Department of Mathematics, Tulane University, New Orleans, LA 70118, USA; dflorez@tulane.edu (D.F.); mhyman@tulane.edu (J.M.H.); 2 School of Public Health and Tropical Medicine, Tulane University, New Orleans, LA 70112, USA; ayoung1@tulane.edu (A.J.Y.); kbernabe@tulane.edu (K.J.B.); 3 Department of Mathematics, University of Texas at San Antonio, San Antonio, TX 78249, USA

**Keywords:** *Anopheles* mosquitoes, *Wolbachia*, malaria control, mosquito control, mathematical model

## Abstract

*Wolbachia* infection in *Anopheles albimanus* mosquitoes can render mosquitoes less capable of spreading malaria. We developed and analyzed a mechanistic compartmental ordinary differential equation model to evaluate the effectiveness of *Wolbachia*-based vector control strategies among wild *Anopheles* mosquitoes in Haiti. The model tracks the mosquito life stages, including egg, larva, and adult (male and female). It also accounts for critical biological effects, such as the maternal transmission of *Wolbachia* through infected females and cytoplasmic incompatibility, which effectively sterilizes uninfected females when they mate with infected males. We derive and interpret dimensionless numbers, including the basic reproductive number and next-generation numbers. The proposed system presents a backward bifurcation, which indicates a threshold infection that needs to be exceeded to establish a stable *Wolbachia* infection. The sensitivity analysis ranks the relative importance of the epidemiological parameters at baseline. We simulate different intervention scenarios, including prerelease mitigation using larviciding and thermal fogging before the release, multiple releases of infected populations, and different release times of the year. Our simulations show that the most efficient approach to establishing *Wolbachia* is to release all the infected mosquitoes immediately after the prerelease mitigation process. Moreover, the model predicts that it is more efficient to release during the dry season than the wet season.

## 1. Introduction

Malaria is a febrile illness caused by several species of *Plasmodium* protozoan parasites and transmitted by the bite of an infected female *Anopheles (An.)* mosquito [1]. Falciparum malaria is a leading cause of death globally and the most lethal of the five known species of *Plasmodium* that can infect humans [2]. Current efforts to control malaria typically focus on strengthening surveillance, administering seasonal malaria chemoprophylaxis, or reducing mosquito populations, through means such as distributing insecticide-treated bed nets, implementing larval control, and conducting indoor residual spraying [3]. Due to increasing insecticide resistance, the impact of climate change, and other environmental factors on mosquito breeding and feeding behavior, more sustainable and effective mitigation strategies will be needed.

*Wolbachia pipientis* is a Gram-negative, intracellular endosymbiotic bacterium that naturally infects over 75% of all arthropods [4,5], including mosquitoes that spread human diseases. Transinfection of *Aedes* spp. mosquitoes is shown to be effective at controlling dengue fever, chikungunya, and Zika virus transmission. Recently, evidence has suggested that similar approaches can control the spread of *P. falciparum* malaria [6,7,8].

Mathematical models have been a great tool to understand infectious disease dynamics and predict mitigation efforts, as they provide an analytical framework to describe and characterize the complex interactions between counterparts in systems. We aim to use our modeling tool to characterize the establishment of *Wolbachia* among the mosquito population as mosquito controls and inform various aspects of designing an effective *Wolbachia* release trial. *Wolbachia* has been used as both a *population suppression* strategy and * population replacement* strategy (Table 1), and our modeling work focuses on scenarios related to the population replacement strategy.

The population suppression strategy involves releasing infected males only. The *Wolbachia*-induced cytoplasmic incompatibility (CI) phenomenon provides an alternative approach similar to adulticide. The sustainability issue and the accidental release of infected females may undermine the process [9,10].

The population replacement strategy involves releasing both male and female infected mosquitoes into the field [11,12]. Infecting *Anopheles* mosquitoes with *wMelPop* and *wAlbB* strains of *Wolbachia* show a reduction in *P. falciparum* sporozoite and oocyst levels in specific species (Table 2) [7], and *Wolbachia*-infected *Anopheles* mosquitoes are less effective in transmitting the parasite. These antipathogenic traits can be passed to offspring since *Wolbachia* exhibits high rates of maternal transmission in both *Aedes* and *Anopheles* spp. mosquitoes [4,6,13]. This leads to a population replacement strategy, where instead of removing the wild mosquitoes, the goal is to infect mosquitoes with *Wolbachia* and replace the wild mosquito population with the infected ones that can no longer transmit the malaria parasite. Field studies show that the population replacement strategy can be a more sustainable approach [14,15]. For our modeling study, we base our parameterizations on the *wAlbB* strain, which exhibits perfect maternal transmission in *An. stephensi* [6,13].

The existing mathematical models for studying the *Wolbachia* infection in mosquitoes primarily focus on arboviruses spread by *Aedes* spp. mosquitoes. Xue et al. [22] compared the impact of infecting *Aedes aegypti* and *Aedes albopictus* mosquitoes with *wAlbB* and *wMel* in reducing the transmission of dengue, chikungunya, and Zika viruses. This study analyzed a system of seven ordinary differential equations (ODEs) that accounted for the reduced fitness of *Wolbachia-*infected mosquitoes, reduced transmissibility of infected mosquitoes, and behavior changes of infected humans caused by disease. This model was based on previous studies that modeled the potential of establishing *Wolbachia* in wild *Aedes* mosquitoes [23,24,25] and incorporated a series of two-sex compartmental models for *Wolbachia* transmission in *Aedes* mosquitoes. These models quantify the effectiveness of different approaches to ensure the sustained transmission of *Wolbachia* within wild *Aedes* mosquitoes.

Our current modeling work is motivated by Qu et al. [23] but with several key differences. We subdivide the mosquito aquatic stage into the egg and larval/pupae stages to emphasize the potential impact of the environment. We also eliminate the impregnated mosquito compartments by assuming the impregnation time is relatively short compared to the lifespan of mosquitoes. Similar to previous studies, we identify a threshold condition for *Wolbachia* replacement, which requires a minimum infection of 34% to be achieved among mosquitoes. In addition, we consider the impact of incorporating malaria-specific interventions before *Wolbachia* releases to accelerate establishing *Wolbachia* infection among mosquitoes. Lastly, we study how the seasonal variation in the environment may impact the deployment of the *Wolbachia* population. Based on the remote sensing data from Grand Anse, Haiti, our model suggests that releasing infection right before the dry season is more efficient.

After defining our compartmental ODE model (Section 2.1), we analyze the model by introducing the next-generation numbers, $G_{0u}$ and $G_{0w}$, for the uninfected and infected mosquito populations (Section 2.2.1). We derive the reproductive number, $R_{0}$, for the spread of *Wolbachia* in the mosquito population and illustrate how $R_{0}$ can be interpreted in terms of the next-generation numbers (Section 2.2.2). We then compare different release scenarios and investigate the effect of concurrent malaria vector control interventions and the impact of seasonality (Section 3).

## 2. Materials and Methods

### 2.1. Model Description

**Figure 1:**
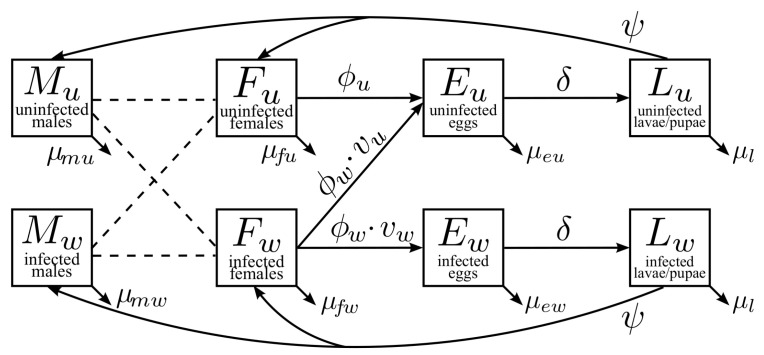
Maternal transmission of *Wolbachia* in mosquitoes. The adult population of males and females is divided into compartments based on the infection status. Uninfected females (*F_u_*) produce uninfected eggs (*E_u_*) at an egg-laying rate of *ϕ_u_*. Infected females (*F_w_*) produce a fraction of *v_w_* infected eggs (*E_w_*) with a rate of *ϕ_w_*. Then, eggs develop into the larval stage at a hatching rate of *δ*. Larvalstage mosquitoes emerge at a rate of *ψ* and develop into adult mosquitoes. Death rates at different stages are denoted by μ_*_.

Our multistage, two-sex model partitioned the mosquito population by life stages and *Wolbachia*-infection status. Figure 1 illustrates the maternal transmission of *wAlbB* in mosquitoes. The adult stages include uninfected males ($M_{u}$), infected males ($M_{w}$), uninfected females ($F_{u}$), and infected females ($F_{w}$). The uninfected eggs ($E_{u}$) and infected eggs ($E_{w}$) are separate compartments. The larvae and pupae stages were combined into one stage for the uninfected ($L_{u}$) and infected groups ($L_{w}$). Model parameters and baseline values for the simulations are shown in Table 3. The details on the parameter estimates are found in Section 2.3.

#### 2.1.1. Male Adult Mosquitoes ($M_{u}$ and $M_{w}$)

Uninfected and infected males have mean lifespans of $\tau_{mu}$ or $\tau_{mw}$. We assumed an exponential survival rate, which led to constant daily death rates of $\mu_{mu}=1/\tau_{mu}$ or $\mu_{mw}=1/\tau_{mw}$. The uninfected and infected males randomly mix and impregnate females. We assumed that *Wolbachia*-infection minimally affected the mating behavior of mosquitoes. Therefore, infected males were nearly as competent as the uninfected males [6]. The probability that a random male mosquito was uninfected or infected was determined by the proportions

(1) $$M_{u}=\frac{M_{u}}{M_{u}+M_{w}},\;\mathrm{and}\;M_{w}=1-M_{u}=\frac{M_{w}}{M_{u}+M_{w}}\;.$$

#### 2.1.2. Female Adult Mosquitoes ($F_{u}$ and $F_{w}$)

*Wolbachia*-infection may affect the lifespan of females [6]. The uninfected and infected females have a mean lifespan of $\tau_{fu}$ and $\tau_{fw}$, respectively, and give constant daily death rates of $\mu_{fu}=1/\tau_{fu}$ and $\mu_{fw}=1/\tau_{fw}$.

For simplification, we assumed that all females were impregnated soon after hatching and did not distinguish between nonpregnant and pregnant females (see [24,25]). This assumption slightly adjusted the average daily egg-laying rates $\phi_{u}$ and $\phi_{w}$ for uninfected and infected females, respectively.

#### 2.1.3. Egg Stages ($E_{u}$ and $E_{w}$)

The fraction of infection among eggs produced by infected females ($v_{w}$) is independent of the infection status of males. This fraction is known as the maternal transmission rate. The remainder of the eggs are uninfected ($v_{u}=1-v_{w}$).

When an uninfected female is impregnated by an infected male ($F_{u}$ cross $M_{w}$, with probability $M_{w}$, as defined in Equation (1)), the *Wolbachia*-induced CI may cause a fraction of the impregnated females to lay nonviable eggs ($c_{i}$). Thus, a fraction $c_{i}M_{w}$ of uninfected females are sterile, and the remainder of the eggs are fertile and produce viable uninfected eggs ($\left(1-c_{i}\right)M_{w}$). Thus, the birth rate of the uninfected eggs is given by

$$M_{u}\phi_{u}+M_{w}\left(1-c_{i}\right)\phi_{u}+M_{w}c_{i}\;\cdot \;0\;.$$

without *Wolbachia*-induced CI ($c_{i}=0$), the birth rate of the viable uninfected is $\phi_{u}$ per day. For the baseline simulations with wAlbB, we assumed a complete CI ($c_{i}=1$), and the birth rate for $E_{u}$ was $\phi_{u}M_{u}$.

Although *Wolbachia*-infection may not affect the total number of offspring that the infected female reproduces [6], it can impact the survivorship of the eggs produced. Thus, we assumed that the uninfected and infected eggs had daily death rates of $\mu_{eu}$ and $\mu_{ew}$. Moreover, we assumed surviving eggs then hatched at a rate of δ regardless of infection status.

#### 2.1.4. Larvae/Pupae Stages ($L_{u}$ and $L_{w}$)

We combined the larvae and pupae stages and limited the population using a logistic carrying-capacity constraint ($K_{l}$). The carrying capacity is dependent on the availability of water and food resources, and it was incorporated into the model by applying the constraint

(2) $$K=1-\frac{L_{u}+L_{w}}{K_{l}}$$

on the birth rate of the larvae/pupae group. The carrying capacity accounts for the seasonal variations that affect mosquito populations. We used the seasonally adjusted carrying capacity to investigate the impact of seasonality on establishing *Wolbachia* by releasing infected mosquitoes (Section 3.4).

The adult mosquitoes emerge from the larvae stages at a constant rate (ψ). The emergence rates are not significantly different between the wild and infected cohort [6]. The fraction of larvae that emerge to become females is denoted as $b_{f}$. The fraction of larvae that emerge to become males is defined as $b_{m}=1-b_{f}$.

These assumptions were satisfied by the solution to the system of differential equations

(3) $$\begin{array}{cc} \frac{dM_{u}}{dt} & =b_{m}\psi L_{u}-\mu_{mu}M_{u}\;,\\ \frac{dM_{w}}{dt} & =b_{m}\psi L_{w}-\mu_{mw}M_{w}\;,\\ \frac{dF_{u}}{dt} & =b_{f}\psi L_{u}-\mu_{fu}F_{u}\;,\\ \frac{dF_{w}}{dt} & =b_{f}\psi L_{w}-\mu_{fw}F_{w}\;,\\ \frac{dE_{u}}{dt} & =\phi_{u}M_{u}F_{u}+\phi_{w}v_{u}F_{w}-\delta E_{u}-\mu_{eu}E_{u}\;,\\ \frac{dE_{w}}{dt} & =v_{w}\phi_{w}F_{w}-\delta E_{w}-\mu_{ew}E_{w}\;,\\ \frac{dL_{u}}{dt} & =\delta KE_{u}-\psi L_{u}-\mu_{l}L_{u}\;,\\ \frac{dL_{w}}{dt} & =\delta KE_{w}-\psi L_{w}-\mu_{l}L_{w}\;. \end{array}$$

Here, $M_{u}$ and K are dimensionless quantities, defined as nonlinear functions of the state variables (Equations (1) and (2)).

**Table 3:** Model parameters and the baseline values. All rates have the unit day${}^{-1}$.

	Description	Value	Reference
	**Specific to** ***Anopheles *** **spp.**		
δ	Hatching rate for eggs (=$1/\tau_{\delta }$)	1/3	[26]
ψ	Emergence rate for larvae (=$1/\tau_{\psi }$)	1/18	[6]
$\mu_{fu}$	Death rate for uninfected females (=$1/\tau_{fu}$)	1/13	[6,26]
$\mu_{fw}$	Death rate for infected females (=$1/\tau_{fw}$)	1/15	[6,26]
$\mu_{mu}$	Death rate for uninfected males (=$1/\tau_{mu}$)	1/7	[6,26]
$\mu_{mw}$	Death rate for infected males (=$1/\tau_{mw}$)	1/7	[6,26]
$\mu_{eu}$	Death rate for uninfected eggs	0.12	[6]
$\mu_{ew}$	Death rate for infected eggs	0.33	[6]
$\mu_{l}$	Death rate for larvae	0.01	[6]
$\phi_{u}$	Per capita egg laying rate for wild females	3.8	[6]
$\phi_{w}$	Per capita egg laying rate for infected females	3.3	[6]
$v_{w}$	*wAlbB* maternal transmission fraction	1	[7]
$c_{i}$	*wAlbB* CI fraction	1	
	**Not specific to ** ***Anopheles *** **spp.**		
$b_{f}$	Fraction of larvae emerging as females	0.5	[27]
$b_{m}$	Fraction of larvae emerging as males	0.5	[27]
$K_{l}$	Carrying capacity of larvae/pupae stages	$2\times 10^{5}$	Assume

### 2.2. Model Analysis

We analyzed the model (Equation (3)) by first defining two *next-generation numbers*, $G_{0u}$ and $G_{0w}$. These factors provided insightful information on mosquito reproduction and reflected the competition between the uninfected and infected cohorts during the population replacement process [23].

#### 2.2.1. Next-Generation Numbers

When there is no *Wolbachia* infection in the population, the average number of uninfected eggs that an uninfected female lays over a lifetime is given by $\phi_{u}/\mu_{fu}$. A fraction $\delta /(\delta +\mu_{eu})$ of these uninfected eggs can survive and develop into the larvae/pupae stage. With probability $b_{f}\;\psi /\left(\psi +\mu_{l}\right)$, larvae develop into uninfected female adults. The product of these factors gives the number of new uninfected females generated by one uninfected female through one generation,

(4) $$G_{0u}=b_{f}\frac{\psi }{\psi +\mu_{l}}\;\frac{\delta }{\delta +\mu_{eu}}\;\frac{\phi_{u}}{\mu_{fu}}\;,$$

which we defined as the next-generation number for the uninfected population. Near the baseline parameter values (Table 3), we had $G_{0u}>1$, indicating that the wild mosquito population can persist when there are no *Wolbachia*-infected mosquitoes.

Similarly, we defined the next-generation number for the infected population,

(5) $$G_{0w}=v_{w}b_{f}\frac{\psi }{\psi +\mu_{l}}\;\frac{\delta }{\delta +\mu_{ew}}\;\frac{\phi_{w}}{\mu_{fw}}\;,$$

where $v_{w}$ is the maternal transmission rate, which gives the fraction of infected eggs produced by the *Wolbachia*-infected females.

#### 2.2.2. Equilibria and Basic Reproductive Number

The model had three types of equilibrium points: disease-free equilibrium, complete-infection equilibrium, and endemic equilibrium.

##### Disease-Free Equilibrium (DFE)

We derived the DFE by setting the populations in all infected stages equal to zero in the system (Equation (3), $E_{w}=L_{w}=F_{w}=M_{w}=0$). The corresponding equilibrium solution gave the DFE denoted by $X^{0}={\left(E_{u}^{0},0,L_{u}^{0},0,F_{u}^{0},0,M_{u}^{0},0\right)}^{T}$,

(6) $$\begin{array}{cc} E_{u}^{0} & =b_{f}\frac{\psi }{\mu_{fu}}\frac{\phi_{u}}{\delta +\mu_{eu}}L_{u}^{0}\;,\\ L_{u}^{0} & =K_{l}\left(1-\frac{1}{G_{0u}}\right)\;,\\ F_{u}^{0} & =b_{f}\frac{\psi }{\mu_{fu}}L_{u}^{0}\;,\\ M_{u}^{0} & =b_{m}\frac{\psi }{\mu_{mu}}L_{u}^{0}\;, \end{array}$$

where $G_{}0u$ is the next-generation number for the uninfected population (4).

##### Complete-Infection Equilibrium (CIE)

The CIE exists when assuming perfect maternal transmission, that is, $v_{w}=1$, and all the mosquitoes are infected. We derived the CIE by setting all the uninfected compartments to zero, i.e., $E_{u}=L_{u}=F_{u}=M_{u}=0$, in the system (Equation(3)), and the corresponding equilibrium solution gave the CIE, which was denoted by $X^{c}={\left(0,E_{w}^{c},0,L_{w}^{c},0,F_{w}^{c},0,M_{w}^{c}\right)}^{T}$,

(7) $$\begin{array}{cc} E_{w}^{c} & =b_{f}\frac{\phi_{w}}{\delta +\mu_{ew}}\;\frac{\psi }{\mu_{fw}}L_{w}^{c}\;,\\ L_{w}^{c} & =K_{l}\left(1-\frac{1}{G_{0w}}\right)\;,\\ F_{w}^{c} & =b_{f}\frac{\psi }{\mu_{fw}}L_{w}^{c}\;,\\ M_{w}^{c} & =b_{m}\frac{\psi }{\mu_{mw}}L_{w}^{c}\;, \end{array}$$

where $G_{0w}$ is the next-generation number for the infected population (5).

##### Basic Reproductive Number

Following the next-generation matrix approach, we considered the infected compartments in the model (Equation (3)), denoted by $X_{w}$ = ($E_{w}$, $L_{w}$, $F_{w}$, $M_{w}$)${}^{T}$, and defined a subsystem for these variables,

$$\frac{dX_{w}}{dt}=\frac{d}{dt}\left(\begin{array}{c} E_{w}\\ L_{w}\\ F_{w}\\ M_{w} \end{array}\right)=F-V=\left(\begin{array}{c} v_{w}\phi_{w}F_{w}\\ 0\\ 0\\ 0 \end{array}\right)-\left(\begin{array}{c} \left(\delta +\mu_{ew}\right)E_{w}\\ -\delta \left(1-\frac{L_{u}+L_{w}}{K_{l}}\right)E_{w}+\left(\psi +\mu_{l}\right)L_{w}\\ -b_{f}\psi L_{w}+\mu_{f_{w}}F_{w}\\ -b_{m}\psi L_{w}+\mu_{mw}M_{w} \end{array}\right)\;,$$

where the vectors F and V represent the rate of new infections and the transition rate among the infected compartments. We then linearized the equation at the DFE and obtained the Jacobian matrices $J_{F}\left(X^{0}\right)$ and $J_{V}\left(X^{0}\right)$,

$$J_{F}=\left(\begin{array}{cccc} 0 & 0 & v_{w}\phi_{w} & 0\\ 0 & 0 & 0 & 0\\ 0 & 0 & 0 & 0\\ 0 & 0 & 0 & 0 \end{array}\right)\;,\;J_{V}=\left(\begin{array}{cccc} \delta +\mu_{ew} & 0 & 0 & 0\\ -\delta /G_{0u} & \psi +\mu_{l} & 0 & 0\\ 0 & -b_{f}\psi & \mu_{fw} & 0\\ 0 & -b_{m}\psi & 0 & \mu_{mw} \end{array}\right)\;,$$

and the basic reproductive number was given by

(8) $$R_{0}:=\mathrm{spectral}\;\mathrm{radius}\;\mathrm{of}\;J_{F}J_{V}^{-1}=v_{w}\frac{\mu_{fu}\phi_{w}\left(\delta +\mu_{eu}\right)}{\mu_{fw}\phi_{u}\left(\delta +\mu_{ew}\right)}\;.$$

To interpret the obtained basic reproductive number, we wrote it as the ratio of the next-generation numbers

$$R_{0}=\frac{G_{0w}}{G_{0u}}=v_{w}\frac{\mu_{fu}\phi_{w}\left(\delta +\mu_{eu}\right)}{\mu_{fw}\phi_{u}\left(\delta +\mu_{ew}\right)}\;.$$

Recall the definition of the next-generation numbers $G_{0u}$ and $G_{0w}$ (Section 2.2.1), which represent the numbers of new offspring reproduced per generation among the uninfected and infected cohorts, assuming the system is near DFE. The ratio of these next-generation numbers is an estimate of the average number of infected offspring generated per infected individual at the DFE.

When $G_{0w}>G_{0u}$ or $R_{0}>1$, the infected mosquitoes reproduce more than the uninfected ones. Therefore, the small infection will spread in the population. In practice, the infected population experiences a fitness cost, and $R_{0}<1$ at baseline. Hence, the naturally uninfected population will wipe out a small introduction of an infected population. Consequently, the ratio of next-generation numbers is a threshold condition for a small initial invasion of the *Wolbachia*-infected population in the wild mosquito population.

##### Endemic Equilibrium (EE)

During imperfect maternal transmission, $v_{w}<1$, some infected females produce uninfected offspring, and the CIE cannot be achieved. Instead, there is EE, where infected and uninfected mosquitoes coexist.

We first defined $r_{wu}$ as the ratio between the infected and uninfected larvae/pupae stages (i.e., $r_{wu}=L_{w}/L_{u}$) which was a key dimensionless quantity in the derivation. Then, EE, denoted by $X^{*}={\left(M_{u}^{*},M_{w}^{*},F_{u}^{*},F_{w}^{*},E_{u}^{*},E_{w}^{*},L_{u}^{*},L_{w}^{*}\right)}^{T}$, could be written in terms of $r_{wu}$ as follows:

(9) $$\begin{array}{cc} M_{u}^{*} & =b_{m}\frac{\psi }{\mu_{mu}}L_{u}^{*}\;,\\ M_{w}^{*} & =r_{wu}b_{m}\frac{\psi }{\mu_{mw}}L_{u}^{*}\;,\\ F_{u}^{*} & =b_{f}\frac{\psi }{\mu_{fu}}L_{u}^{*},\\ F_{w}^{*} & =r_{wu}b_{f}\frac{\psi }{\mu_{fw}}L_{u}^{*}\;,\\ E_{u}^{*} & =\frac{b_{f}\psi }{\delta +\mu_{eu}}\left(\frac{\phi_{u}}{\mu_{fu}}\frac{1}{1+r_{wu}}+\frac{v_{u}\phi_{w}}{\mu_{fw}}r_{wu}\right)L_{u}^{*}\;,\\ E_{w}^{*} & =r_{wu}\frac{b_{f}\psi }{\delta +\mu_{ew}}\frac{v_{w}\phi_{w}}{\mu_{fw}}L_{u}^{*}=r_{wu}E_{u}^{*}\;,\\ L_{u}^{*} & =\frac{1}{1+r_{wu}}K_{l}\left(1-\frac{1}{G_{0w}}\right)\;,\\ L_{w}^{*} & =r_{wu}L_{u}^{*}\;. \end{array}$$

We assumed the death rate for uninfected males and infected males were the same ($\mu_{mu}=\mu_{mw}$). The ratio $r_{wu}$ satisfies the quadratic relation that involves the maternal transmission rate, infection leakage rate, and basic reproductive number,

(10) $$\frac{v_{u}}{v_{w}}\frac{\delta +\mu_{ew}}{\delta +\mu_{eu}}r_{wu}^{2}+\left(\frac{v_{u}}{v_{w}}\frac{\delta +\mu_{ew}}{\delta +\mu_{eu}}-1\right)r_{wu}+\frac{1-R_{0}}{R_{0}}=0\;.$$

Under the special case of perfect maternal transmission ($v_{w}=1$), Equation (10) degenerates to a linear relation,

$$r_{wu}^{*}=\frac{L_{w}^{*}}{L_{u}^{*}}=\frac{1-R_{0}}{R_{0}}\;.$$

To have a physically relevant endemic equilibrium, we needed to impose $r_{wu}^{*}>0$. This implied that $R_{0}$ had to be between 0 and 1. Our baseline estimate for $R_{0}$ was 0.68.

#### 2.2.3. Stability and Bifurcation Analysis

The stability analysis of the equilibrium points helps to characterize the solution dynamics, and it indicated a threshold condition for establishing stable *Wolbachia*-infection among mosquitoes.

The stability of an equilibrium is determined by the signs of the eigenvalues of the Jacobian matrix for the system of ODEs linearized about the equilibrium. We present the conclusions on the stability analysis below. The proofs of Theorems 1 and 2 are in Appendix A and Appendix B. We numerically verify the conjecture Theorem 3. These conclusions are comparable to the ones in [23], which has a similar model structure.

**Theorem 1**  (Stability of the Disease-free Equilibrium). *The DFE (Equation (6)) of system (Equation (3)) is locally asymptotically stable provided that $G_{0u}>1$ and $R_{0}<1$.*

**Theorem 2**  (Stability of the Complete Infection Equilibrium). *The CIE (Equation (7)) of the system (Equation (3)) is locally asymptotically stable provided that $G_{0w}>1$.*

**Theorem 3**  (Stability of the Endemic Equilibrium). *When having the perfect maternal transmission ($v_{w}=1$), the physically relevant EE (Equation (9)) of system (Equation (3)) exists for $R_{0}<1$ and $G_{}0w>1$, and it is an unstable equilibrium.*

**Figure 2:**
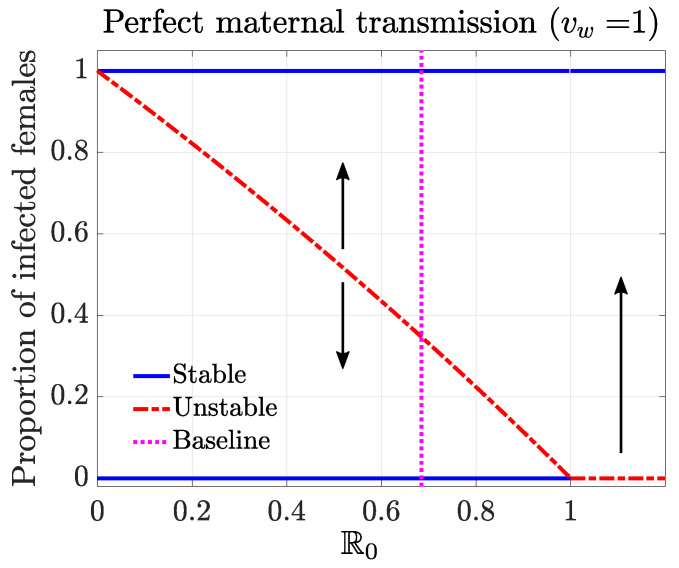
Bifurcation diagram characterizing the threshold condition for establishing a stable infection in mosquitoes, given a perfect maternal transmission rate ($v_{w}=1$). The solid blue curves represent the stable equilibrium. The red dashed curve corresponds to the unstable equilibrium, which serves as the threshold condition. At the baseline case (vertical dotted line, $R_{0}=0.68$), the threshold infection rate among females was 0.34.

Based on the conclusions above, we generated the bifurcation plot (Figure 2). We varied the parameter $\phi_{u}$, while keeping other parameters at the baseline values to calculate the $R_{0}$ values and trace out different steady states. The stability of the steady states led to the backward bifurcation behavior, which highlighted a critical threshold condition for establishing *Wolbachia* infection among mosquitoes over a range of $R_{0}$ values. For example, for a given $R_{0}$, we identified the minimum fraction of infection that needed to be exceeded in females to establish a stable endemic state of *Wolbachia*. Here, we assumed a natural distribution of infection among the population. Above that threshold, the system will approach the complete-infection stable equilibrium. Below the threshold, the system will approach the disease-free stable equilibrium. This threshold infection rate was 34% among females for the baseline case, where $R_{0}=0.68$.

The backward bifurcation behavior can be interpreted as the result of the competition between the infected and uninfected mosquito cohorts. Recall that $R_{0}$ is defined as the ratio between the next-generation factors for uninfected $G_{0u}$ and infected $G_{0u}$, and we describe below two parameter regimes that lead to different competition results between the infected and uninfected mosquito cohorts.

When $R_{0}>1$ ($G_{0w}>G_{0u}$), there is an unstable DFE, which means that the introduction of one infected mosquito will cause a rapid spread of *Wolbachia* among the population. However, under the biologically relevant regime, since the *Wolbachia* infection affects the fitness of the infected mosquitoes, the reproduction $G_{0w}<G_{0u}$ or $R_{0}\leq 1$, and the introduction of a small fraction of infection at DFE will die out. This scenario is consistent with the fact that *Wolbachia* infection is not naturally found in wild *Anopheles* mosquitoes.

Since infected males sterilize uninfected females, if we increase the fraction of infected mosquitoes, the cytoplasmic incompatibility decreases the fitness of the uninfected mosquitoes. As more infected mosquitoes are released, the fitness of the uninfected mosquitoes becomes less than the infected mosquitoes. The fraction where the two fitness values are the same corresponds to the threshold condition for sustaining the endemic *Wolbachia* infection.

### 2.3. Parameter Estimations

Most of our estimates were based on work by Joshi et al. [6], which characterized the life parameters of the mosquitoes in an ideal lab setting. These parameters can vary depending on environmental factors. In Section 3.4, we discuss the impact of seasonality on *Wolbachia*-releasing strategies.

#### 2.3.1. Maternal Transmission

Maternal transmission from infected females to their offspring is the primary mechanism by which *Wolbachia* is transmitted to other mosquitoes. *wAlbB*-infected females have close to perfect (100%) maternal transmission where almost all of the offspring of infected females are infected.

#### 2.3.2. Mosquito Lifespan

*Wolbachia*-infection has a minor, if any, impact on the longevity of males. The median lifespan, under a laboratory condition, is about 16 days [6]. However, in a competitive field environment, the lifespan is shorter [26]; thus, we used the realistic estimate for the lifespan of male mosquitoes ($\tau_{mu}=\tau_{mw}=7$ days). When fed on human blood, infected and uninfected females live for approximately 22 days in the lab. However, the infected females show better survivorship during the first two weeks [6]. Thus, we assumed a shorter lifespan in the realistic setting, $\tau_{fu}=13$ days and $\tau_{fw}=15$ days.

#### 2.3.3. Egg-laying Rates

*Wolbachia* infection has a negligible impact on the total number of eggs an *Anopheles* female mosquito produces throughout its lifetime, which is about 50 eggs/female [6]. Thus, the daily egg-laying rates for the uninfected females ($\phi_{u}$) is $50/\tau_{fu}\approx 3.8$ eggs/day and $50/\tau_{fw}\approx 3.3$ eggs/day for the infected females ($\phi_{w}$).

#### 2.3.4. Egg-hatching Rate and Death Rates

We assumed that the *Wolbachia* infection did not impact the egg-hatching period. On average, it takes about three days for eggs to hatch [26]; thus, we set $\tau_{\delta }=3$ days and $\delta =1/\tau_{\delta }=1/3$. *Wolbachia* infection reduces the fecundity in female mosquitoes; the fraction of eggs that hatch and survive to first-instar larvae is lower among the infected population than the uninfected one (50% vs. 73%) [6]. Thus, we had $\delta /(\delta +\mu_{eu})=0.73$ and $\delta /(\delta +\mu_{ew})=0.5$, which yielded death rate estimates of $\mu_{eu}\approx 0.12$ and $\mu_{ew}\approx 0.33$ for eggs.

#### 2.3.5. Larvae/Pupae Emergence Rate and Death Rate

*Wolbachia* has no significant impact on the life traits of *Anopheles* mosquitoes, including the emergence time from larvae to adults and survivorship. On average, it takes about $\tau_{\psi }=18$ days (sum of pupation time and emergence time, see [6]), which gives the emergence rate $\psi =1/\tau_{\psi }=1/18$. The fraction of larvae that survive to the adult stage is $\psi /(\psi +\mu_{l})\approx 80\%$; thus, the daily death rate for the larvae/pupae stage was estimated as $\mu_{l}=0.01$.

## 3. Results

Our numerical simulations aimed to provide qualitative insights for designing optimal release strategies to establish a stable *Wolbachia* infection in mosquito populations. We compared prerelease larvicide and thermal fogging, releasing multiple batches of *Wolbachia* mosquitoes, and the time of year for the release.

### 3.1. Sensitivity Analysis

We quantified the significance of the parameters in the model predictions using a local sensitivity analysis. This helped us better understand our model when parameters were changed.

We used the normalized relative sensitivity index of a quantity of interest (QOI), q(p), with respect to a parameter of interest (POI), *p*, defined as $S_{p}^{q}:=\frac{p}{q}\times \frac{\partial q}{\partial p}$. This index measured the percentage change in the QOI given the percentage change in the input POI. In other words, if parameter *p* changed by α%, then *q* would change by $S_{p}^{q}\times \alpha \%$. The sign determined the decreasing or increasing behavior of the quantity. We evaluated the index at the baseline parameter values to obtain the local sensitivity index.

**Figure 3:**
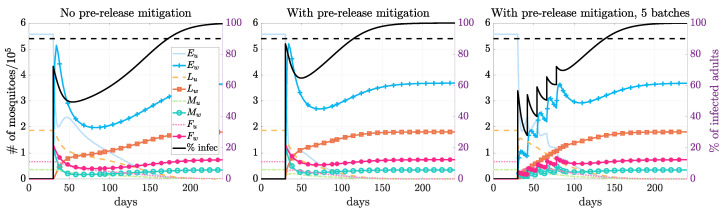
Simulations for different release scenarios. The left figure displays the mosquito populations for a single release of infected mosquitoes when there is no prerelease mitigation. The middle and right figures compare releasing all the mosquitoes in a single release and in multiple batches when there is prerelease mitigation. An equal number of infected male and female mosquitoes are released (release size = 2, relative to baseline female population size at DFE) without or with prerelease mitigation (reduced to 40% in both larvae and adults using hybrid fogging and larviciding, see Table 5). The black line is the percent of infected mosquitoes that are infected as they are released in one batch or multiple batches. The *Wolbachia* endemic state is established, and the infection reaches 90% infection around 143, 85, and 109 days after the initial release.

We considered three different QOIs concerning the establishment of *Wolbachia* infection: the reproductive number $R_{0}$; the threshold of infection in females, which corresponded to the unstable equilibrium indicated in the bifurcation diagram of Figure 2; and the establishment time, measured as the time to achieve 90% infection for a particular release setting of interest (Figure 3, with prerelease mitigation and released in five batches).

The sensitivity indices were ranked by magnitude (importance) for the QOI = threshold case in Table 4. Following this criterion, the maternal transmission rate $\nu_{w}$ was the most sensitive parameter among all the selected POIs. In addition, the egg-laying rates ($\phi_{u}$ and $\phi_{w}$) and the adult mosquito lifespans of females ($\mu_{fw}$ and $\mu_{fu}$) also had a significant sensitivity with respect to the QOIs. That is, the parameters related to the reproduction and CI of mosquitoes were critical to both the threshold and the speed of establishing a sustained *Wolbachia* infection.

Conversely, parameters involved in the survival of eggs, such as the egg lifespans ($\mu_{eu}$ and $\mu_{ew}$) and their hatching rate δ, were less sensitive in the three QOIs studied. Furthermore, due to the assumption $\mu_{eu}=\mu_{ew}$, the adult male lifespans ($\mu_{mu}$ and $\mu_{mw}$) did not represent an impact in $R_{0}$ and the threshold condition. Instead, they played a relevant role in the time to establish a sustained infection once infection exceeded the threshold.

We also simultaneously perturbed all the adult death rates $\mu_{fu}$, $\mu_{fw}$, $\mu_{mu}$, and $\mu_{mw}$. This simulated a change in the global environment that affected both infected and uninfected mosquitoes. As indicated by the $\mu_{adults}$ column, this change did not have a significant impact on $R_{0}$ and the threshold. This can be understood by checking the individual sensitivity indices for $\mu_{fw}$ and $\mu_{fu}$, the perturbation of which gave the same amount of change in opposite directions. Thus, the simultaneous change neutralized the impact and did not affect the competition outcome. Meanwhile, the change did delay the establishment process, as the infected cohort ($\mu_{fw},\mu_{mw}$) had a larger impact on the establishment time.

### 3.2. Compare Prerelease Mitigation Strategies

To reduce the number of infected mosquitoes released and more efficiently establish a stable *Wolbachia* infection, integrated control strategies are often implemented to reduce the wild mosquito population before releasing the infected mosquitoes. We evaluated the establishment of *Wolbachia* when combining prerelease mitigation approaches, including larviciding and thermal fogging. Not all of those strategies are primary vector control interventions in Haiti. Nevertheless, our results can inform the potential effectiveness should such interventions become prevalent in the area.

*Larviciding* treats mosquito breeding sites with bacterial or chemical insecticides to kill the aquatic stage of mosquitoes. Field studies of bacterial larvicide products, targeting *Anopheles* larval habitats, report a larval reduction between 47% and 100% [28]. Our model simulated a range of mitigation efficacy (in reducing population) from a more challenging setting of 0.2 to a high efficacy of 0.6.

*Space spraying* or *thermal fogging* refers to dispersing a liquid fog of insecticide into an outdoor area to kill adult insects. The insecticide may be delivered using hand-held, vehicle-mounted, or aircraft-mounted equipment [29]. The impact of fogging as a malaria vector control intervention for reducing adult *Anopheles* mosquitoes fluctuates between 50∼100% [30], and we evaluated the impact of thermal fogging for a moderate range of efficacy, where the mitigation efficacy varied from 0.2∼0.6.

As summarized in Table 5, starting from the baseline DFE state, we simulated the prerelease mitigation (column 1) at different intensities by adjusting the DFE according to the mitigation efficacy at the targeting stage(s) (columns 2–3). We assumed that the prerelease mitigations only impacted the wild mosquitoes and not the released infected mosquitoes. We then released an equal number of infected males and females, and we identified the threshold quantity (needed for establishing the *Wolbachia* endemic state) without a time limit (column 4) and with a time limit of two months (column 5). The release size was quantified using the *release factor*, which is the ratio between the number of released mosquitoes and the number of females at DFE, i.e., $F_{u}^{0}$ in Equation (6).

With prerelease mitigation, a larger mitigation efficacy reduced the release factor for both thermal fogging and larviciding. This was expected since the threshold was determined by the competition (or ratio) between the infected and uninfected cohorts. Fewer infected mosquitoes were needed to match the competition if there were fewer uninfected mosquitoes in the field.

Under the same release size, prerelease mitigation helped to speed up the establishment of the *Wolbachia* endemic equilibrium (Figure 3). We also saw that thermal fogging required a slightly smaller release size than larviciding under the same intervention intensity. When applying two interventions together, it outperformed the individual case as expected. When releasing just above the threshold, it may take a long time to establish a *Wolbachia* endemic state. The identified threshold value may not be practical due to various model assumptions, such as seasonality. When imposing a two-month time limit, many more infected mosquitoes must be released. Such a large release size may not be practical for field trial implementation; thus, we study the multiple-release strategy next.

### 3.3. Multiple Releases

**Figure 4:**
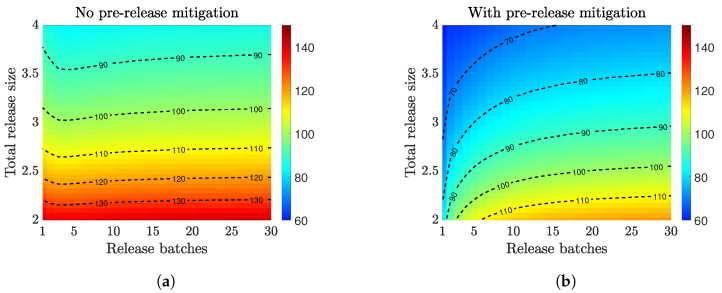
Compare multiple-release strategy for *Wolbachia* establishment speed without and with prerelease mitigation strategies. The heatmaps indicate the days to achieve 90% infection (color-coded according to the respective color bars). The release size (y-axis) is measured relative to the baseline female population size at DFE, and an equal number of infected males and females are released. When no prerelease mitigation is implemented (**a**), an optimal number of release batches is observed for large releases sizes. When prerelease mitigation is implemented (**b**), releasing all infected mosquitoes at once is more efficient than splitting them into multiple batches.

Field trials often require periodic releases of batches of infected mosquitoes. We aimed to inform an optimal design of a multiple release strategy. We considered a certain number of mosquitoes (release size) split into multiple batches (release batches) released over two months. We assumed all the releases had the same number of infected mosquitoes. That is, each batch of released mosquitoes was the total release size divided by the number of batches, and they were released at regular time intervals. In Figure 4, we plotted the time to achieve 90% infection when using different numbers of release batches and total release sizes, and we studied how the establishment time was impacted when using the prerelease mitigation.

For both scenarios, using a larger release size always helped to speed up the establishment of *Wolbachia* infection for both release scenarios. Without prerelease mitigation (Figure 4a), the optimal multiple-release strategy left about a two-week gap (four or five batches within two months) between two consecutive releases. The benefit of such a release gap was more significant as the overall release size increased. The necessity of the release gap resulted from the limited environmental resources available (carrying capacity). Releasing all the infections at once may not be as optimal as splitting the release into multiple batches due to the higher penalization from the carrying capacity. Nonetheless, using too many batches decreased the invasion efficiency.

On the other hand, when there was prerelease mitigation (Figure 4b), it created a gap in the carrying capacity. This gap provided an opportunity for instant population replacement by the infected cohort. Thus, releasing infected mosquitoes all at once was more efficient than splitting the release of infection in batches.

### 3.4. Seasonality

Environmental and climactic covariates, such as rainfall and temperature, affect all the stages of the mosquito life cycle. They impact the density and distribution of vector breeding sites, the number of eggs laid, the ability of larvae to emerge from eggs once they are laid (hatching or emergence rate), and the adult mosquito lifespan. Regional carrying capacity is also affected as this parameter is directly influenced by the number of available vector breeding and egg-laying sites. It is important to account for those seasonality effects by adjusting parameters as these values influence the ability to achieve endemic, stable *wAlbB* transmission among the mosquito population.

We extracted the Climate Hazards Group InfraRed Precipitation with Station (CHIRPS) monthly data for the department of Grand Anse [31] in Haiti, where most of the country’s malaria transmission occurs. In particular, we considered the seasonality pattern based on the rainfall, humidity, and temperature data. We include a summary of the data we used in Table A1.

**Figure A1:**
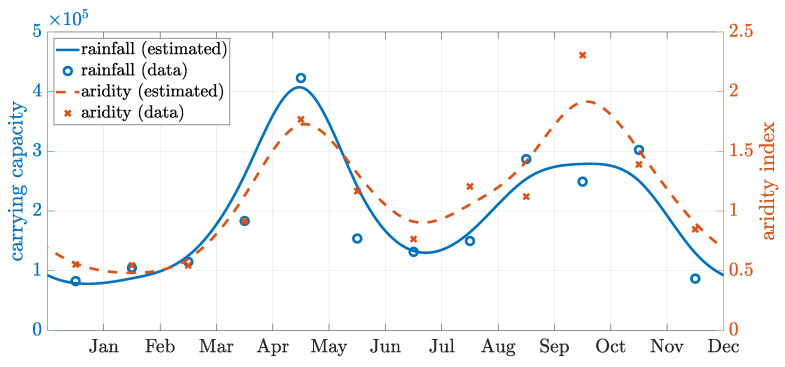
Fitted seasonality curves. Left axis: rescaled rainfall data and the fitted carrying capacity curve (solid blue line). Right axis: aridity index data and the fitted curve (red dashed line). The raw data values listed in Table A1 and points have been shifted to the center of the month. Both estimated curves are fitted using a periodic smoothing spline, showing a consistent trend.

The monthly rainfall data suggested a bimodal seasonal pattern with the peak rainfall in May and September (Figure A1). Therefore, we adapted our model to a time-dependent carrying capacity, $K_{l}\left(t\right)$, which varied according to a fitted seasonality curve based on the rainfall data. We also simulated release scenarios starting in the dry or rainy season. There was a similar seasonal trend in the humidity data (Figure A1), measured by the aridity index, with most of the year classified as humid. We captured the impact of humidity by using the same time-varying carrying capacity curve above, and we assumed that it did not impact other life traits of mosquitoes.

The monthly temperature data ranged from 25.7 to 29.8 degrees Celsius (78.2 to 85.7 degrees Fahrenheit). Temperature can influence egg laying rates, larval emergence rates, and adult mosquito lifespan; however, mean monthly temperature in our region of focus did not vary enough to influence rates for these parameters in our model [32,33].

We aimed to study the seasonality’s impact on field releases’ efficacy in establishing a *Wolbachia* infection. For this purpose, we considered releasing an equal number of female and male infected mosquitoes with a release factor of one (as defined in Table 5), relative to the uninfected female population at the DFE on day 1 of the year, i.e., $F_{u}^{0}$). In addition, the total quantity was released in five batches.

**Figure 5:**
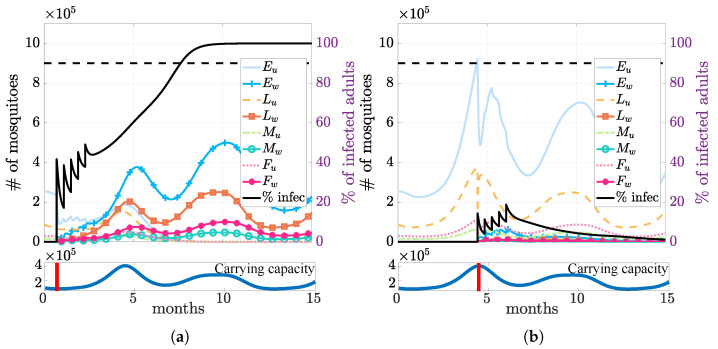
Impact of seasonality on the field release. Simulations of releasing the same number of infected mosquitoes at the driest (day = 21, (**a**)) vs. the wettest (day = 134, (**b**)) time of the year, indicated by the red vertical bars on the corresponding lower panels. The black lines for the percent of infected mosquitoes show that the infection is successfully established when released in the dry season, while it dies out when released in the wet season.

Under the above setting, our simulation results suggested that it was more efficient to establish *Wolbachia* during the dry season. Releasing infection during the dry season (Figure 5a) required releasing fewer infected mosquitoes to exceed the threshold. In contrast, when releasing the same number of mosquitoes during the wet season (Figure 5b), the infection failed to establish itself due to the abundance of wild mosquitoes (higher carrying capacity).

## 4. Discussion and Conclusions

We developed and analyzed a compartmental ODE model to describe the establishment of *Wolbachia* infection in wild *Anopheles* mosquitoes. The model tracked male and female mosquitoes through the egg, larval, and adult stages. The model accounted for maternal transmission of *Wolbachia*, cytoplasmic incompatibility, and fitness cost induced from *Wolbachia* infection. Moreover, we incorporated carrying capacity constraints on mosquito population size to study the impact of seasonality, specific to Haiti.

Our model presented a similar analytical behavior to other *Wolbachia* models with similar modeling structures. The basic reproductive number $R_{0}$ was derived and written as the ratio of the two next-generation numbers [23,24,25], $G_{0u}$ and $G_{0w}$, which corresponded to the number of new offspring reproduced per generation for the uninfected and infected mosquitoes. The stability analysis of the model gave a backward bifurcation, where an unstable endemic equilibrium separated a disease-free equilibrium and a stable complete-infection equilibrium. This was also observed in previous *Wolbachia* modeling studies [23,24,25,34,35] as well as in epidemic models for different diseases [36]. The bistability of the system identified a threshold infection rate that needed to be exceeded to establish a stable *Wolbachia* infection. This observation supports what has been reported in field trials [12] and mosquito cage experiments [37].

Our numerical simulations on *Wolbachia* releases with prerelease mitigations showed that the prerelease mitigations reduced the number of infected mosquitoes needed to exceed the threshold condition and accelerated the establishment of *Wolbachia* infection. In particular, we analyzed baseline mitigation strategies using larviciding and thermal fogging to reduce the wild mosquito population. These approaches do not reflect all the current interventions in Haiti [38], such as insecticide-treated nets [39], which require coupling the current mosquito model with human hosts. These need to be considered in future studies when estimating the impact of the *Wolbachia*-based strategy on malaria transmission among human populations.

We also numerically investigated the impact of seasonality by varying the mosquito population’s carrying capacity. Our simulation results indicated that releasing *Wolbachia*-infected mosquitoes in the dry season was more effective than in the wet season when fewer uninfected mosquitoes were in the wild for competition. This observation agrees with a previous statistical study [40] as well as a field study [41]. We note that mathematical models simplify field conditions, and this conclusion (and many others) depends on our model parametrizations. While incorporating seasonality by only varying carrying capacity is an appropriate approximation for Haiti’s mild variation in temperature and humidity, a more complex model would be necessary for studying locations where seasonality is more prominent.

As in many modeling studies, there are major model limitations related to parametrization. First, our modeling results are only valid for the *wAlbB* strain of *Wolbachia*, and we assumed perfect maternal transmission and *Wolbachia*-induced CI. Parameter values would differ for other strains where these two assumptions do not hold. Moreover, the sparse publication of parameter values for * Wolbachia*-infection in African *Anopheles* vectors results in large uncertainties in our parameter estimates. Most of our baseline parameters were based on Joshi et al. [6], which characterized the life parameters of the mosquitoes in an ideal lab setting. We employed a sensitivity analysis to identify sensitive parameters for various quantities related to the *Wolbachia* establishment. We found that the maternal transmission rate was the most sensitive parameter to all the quantities considered. The other sensitive parameters included egg-laying rates and the lifespans of adult females. Thus, additional studies from lab and field settings on these parameters for both infected and uninfected cohorts will reduce the potential bias in our conclusions.

Our ODE model assumed that the fraction of infection among mosquitoes was homogeneous in space. However, this may not hold in field settings, especially when modeling a local release of infected mosquitoes. Therefore, it is essential to include the impact of spatial dynamics to determine the threshold condition for field releases. We are developing a partial differential equation (PDE) model to study the invasion dynamics of *Wolbachia* infection among mosquitoes in a more realistic field setting. This reaction–diffusion-type model accounts for complex maternal transmission and spatial mosquito dispersion. Our initial studies identified an optimal bubble-shaped distribution to minimize the number of mosquitoes needed to exceed the threshold conditions [42].

## Figures and Tables

**Table 1:** Role of *Wolbachia* infection in mosquito population replacement versus population suppression. CI = cytoplasmic incompatibility.

	Population Replacement	Population Suppression
Goal	Replace wild mosquito population with *Wolbachia*-infected mosquitoes that have significantly lower competence and cannot transmit parasite as efficiently	Introduce male mosquitoes that cannot produce viable offspring, which limits the ability of the mosquito to reproduce and reduces mosquito population [8]
Role of CI	Infected females can mate successfully with infected males providing them with an evolutionary advantage over uninfected females	The sperm of the infected male is unable to form viable offspring during the egg fertilization process, and as a result, eggs do not hatch
Release	Release infected males and females	Release infected males only

**Table 2:** *Wolbachia* strains, *Anopheles* species, and corresponding impact on vector and *P. falciparum* parasite replication. CI = cytoplasmic incompatibility.

*Wolbachia* Strain	*Anopheles* Species	Impact on Vector	Impact on *P. falciparum*	Reference
wAnga	*coluzzii*	No CI, increases egg laying rate	Reduces sporozoite prevalence	[16,17,18,19]
	*funestus*	No CI	Unknown	[18]
	*gambiae*	No CI	Unknown	[18]
	*arabiensis*	No CI	Unknown	[18]
wAlbB	*stephensi*	Almost complete CI, reduces egg hatching rate, perfect maternal transmission, no impact on female lifespan	Reduces sporozoite and oocyst levels	[6,7]
wPip	*gambiae*	CI, reduces egg development rate	Unknown	[20]
wMelPop	*gambiae*	No effect on lifespan	Significantly reduces oocyst level	[21]

**Table 4:** Sensitivity Analysis. Sensitivity indices for threshold-related quantities (in the first column) with respect to the model parameters (first row). Threshold (row 3) refers to the threshold level of infection and the time (row 4) measures the time to achieve 90% infection.

	$\nu_{w}$	$\phi_{w}$	$\phi_{u}$	$\mu_{\mathrm{fw}}$	$\mu_{\mathrm{fu}}$	$\mu_{\mathrm{ew}}$	$\mu_{\mathrm{eu}}$	δ	ψ	$\mu_{\mathrm{mu}}$	$\mu_{\mathrm{mw}}$	$\mu_{l}$	$\mu_{\mathrm{adults}}$
$R_{0}$	1	1	−1	−1	1	−0.5	0.26	0.23	0	0	0	0	−2.4 × 10^−13^
Threshold	−3.5	−2.1	2.1	1.4	−1.4	1	−0.55	−0.48	0	0	0	0	4 × 10^−13^
Time	−6.7	−1.1	1.1	0.88	−0.6	0.51	−0.32	−0.32	−0.6	−0.69	0.6	−0.14	0.14

**Table 5:** Comparison of prerelease mitigation strategies targeting different mosquito life stages (larvae and adults). “Mitigation efficacy” measures the fraction of population reduced given the mitigation approach, and the “release factor” measures the release size of the infected males and females relative to the baseline female population size at DFE ($F_{u}^{0}$). Threshold release sizes needed to establish *Wolbachia* within (two months) or without time constraints are identified.

Prerelease Mitigation	Larvae Mitigation Efficacy	Adults Mitigation Efficacy	Threshold Release Factor	Release Factor to Reach 90% by Two Months
No mitigation (DFE)	0	0	1.13	9.9
Thermal fogging	0	0.2	1.03	9.2
	0	0.4	0.93	9.2
	0	0.6	0.82	7.9
Larviciding	0.2	0	1.04	8.0
	0.4	0	0.96	6.5
	0.6	0	0.88	5.3
Thermal fogging + larviciding	0.6	0.2	0.79	4.8
	0.6	0.4	0.69	4.4
	0.6	0.6	0.60	3.9

## Data Availability

Not applicable.

## Appendix A. Proof of Theorem 1 (Stability of DFE)

Let us consider the variable vector containing the compartments (rearranged by the infection status), $Y=(E_{u},L_{u},F_{u},M_{u},E_{w},L_{w},F_{w},M_{w})$, then the corresponding Jacobian, denoted by *J*, of the system $\frac{dY}{dt}=JY$, is given by

$\;J=\left(\begin{array}{cccccccc} -\delta -\mu_{eu} & 0 & M_{u}\phi_{u} & 0 & 0 & 0 & v_{u}\phi_{w} & 0\\ \delta K & -\frac{\delta E_{u}+K_{l}\left(\mu_{l}+\psi \right)}{K_{l}} & 0 & 0 & 0 & -\frac{\delta }{K_{l}}E_{u} & 0 & 0\\ 0 & b_{f}\psi & -\mu_{fu} & 0 & 0 & 0 & 0 & 0\\ 0 & b_{m}\psi & 0 & -\mu_{mu} & 0 & 0 & 0 & 0\\ 0 & 0 & 0 & 0 & -\delta -\mu_{ew} & 0 & v_{w}\phi_{w} & 0\\ 0 & -\frac{\delta }{K_{l}}E_{w} & 0 & 0 & \delta K & -\frac{\delta E_{w}+K_{l}\left(\mu_{l}+\psi \right)}{K_{l}} & 0 & 0\\ 0 & 0 & 0 & 0 & 0 & b_{f}\psi & -\mu_{fw} & 0\\ 0 & 0 & 0 & 0 & 0 & b_{m}\psi & 0 & -\mu_{mw} \end{array}\right)\;.$

At the disease-free equilibrium, the Jacobian becomes

(A1) $$J_{DFE}=\left(\begin{array}{cc} A_{DFE} & *\\ 0 & D_{DFE} \end{array}\right)\;,\;\mathrm{where}$$

(A2) $$A_{DFE}\;=\;\;\left(\begin{array}{cccc} -\delta -\mu_{eu} & 0 & \phi_{u} & 0\\ \frac{\delta }{G_{0u}} & -G_{0u}\left(\mu_{l}+\psi \right) & 0 & 0\\ 0 & b_{f}\psi & -\mu_{fu} & 0\\ 0 & b_{m}\psi & 0 & -\mu_{mu} \end{array}\right)=\left(\begin{array}{cc} A_{s1} & 0\\ {}* & -\mu_{mu} \end{array}\right)\;,$$

and

(A3) $$D_{DFE}=\left(\begin{array}{cccc} -\delta -\mu_{ew} & 0 & v_{w}\phi_{w} & 0\\ \frac{\delta }{G_{0u}} & -\mu_{l}-\psi & 0 & 0\\ 0 & b_{f}\psi & -\mu_{fw} & 0\\ 0 & b_{m}\psi & 0 & -\mu_{mw} \end{array}\right)=\left(\begin{array}{cc} D_{s1} & 0\\ {}* & -\mu_{mw} \end{array}\right)\;.$$

Observe first that the matrix $J_{DFE}$ is an upper triangular block matrix, and consider the two 4×4 diagonal elements $A_{DFE}$ and $D_{DFE}$ (Equation (A1)). Thus, the stability of the matrix $J_{DFE}$ is equivalent to showing the stability for both matrices.

To show the stability of $A_{DFE}$, notice that it is a lower triangular block matrix with $-\mu_{mu}<0$ (Equation (A2)), thus we just need to consider the 3×3 leading principal submatrix $A_{s1}$, which can be further partitioned as follows,

$$A_{s1}=\left(\begin{array}{ccc} -\delta -\mu_{eu} & 0 & \phi_{u}\\ \delta /G_{0u} & -G_{0u}\left(\mu_{l}+\psi \right) & 0\\ 0 & b_{f}\psi & -\mu_{fu} \end{array}\right)=\left(\begin{array}{cc} A_{1} & B_{1}\\ C_{1} & D_{1} \end{array}\right)\;.$$

To prove the stability of the matrix $A_{s1}$, we use a result on Metzler matrices stated in Proposition 3.1 in [43]. Since $A_{s1}$ is a Metzler matrix, $A_{s1}$ is Metzler stable if and only if $A_{1}$ and $D_{1}-C_{1}A_{1}^{-1}B_{1}$ are Metzler stable. With this in mind, observe that since the matrix $A_{1}$ is lower triangular, its eigenvalues are its corresponding diagonal entries $-(\delta +\mu_{ew})$ and $-G_{0u}\left(\mu_{l}+\psi \right)$, which are both negative. This implies that $A_{1}$ is Metzler stable. Meanwhile, the matrix $D_{1}-C_{1}A_{1}^{-1}B_{1}$ satisfies the property:

(A4) $$D_{1}-C_{1}A_{1}^{-1}B_{1}=\mu_{fu}\left(\frac{1}{G_{0u}}-1\right)<0,\;\;\mathrm{if}\;\;G_{0u}>1\;.$$

Thus, when $G_{0u}>1$, $A_{s1}$ is Meltzer stable, so is the matrix $A_{DFE}$.

Similarly, we derive the condition for the stability of $D_{DFE}$. Given it is a lower triangular block matrix with $-\mu_{mw}<0$ (Equation (A3)), we are left with the 3×3 leading submatrix

$$D_{s1}=\left(\begin{array}{ccc} -\delta -\mu_{ew} & 0 & v_{w}\phi_{w}\\ \delta /G_{0u} & -(\mu_{l}+\psi ) & 0\\ 0 & b_{f}\psi & -\mu_{fw} \end{array}\right)=\left(\begin{array}{cc} A_{2} & B_{2}\\ C_{2} & D_{2} \end{array}\right)\;.$$

By the same argument used previously, $A_{2}$ is Metzler stable and $D_{2}-C_{2}A_{2}^{-1}B_{2}$ satisfies the inequality:

(A5) $$D_{2}-C_{2}A_{2}^{-1}B_{2}=\mu_{fw}\left(R_{0}-1\right)<0,\;\;\mathrm{if}\;\;R_{0}<1\;.$$

In consequence, when $R_{0}<1$, $D_{s1}$ is Metzler stable, so is the matrix $D_{DFE}$.

Finally, combining the two conditions Equations (A4) and (A5) for the stability of $A_{DFE}$ and $D_{DFE}$, we conclude that $J_{DFE}$ is stable provided that $G_{0u}>1$ and $R_{0}<1$. □

## Appendix B. Proof of Theorem 2 (Stability of CIE)

At the complete infection equilibrium, the Jacobian has the form:

$$\;J_{\mathrm{CIE}}=\left(\begin{array}{cccccccc} -\delta -\mu_{eu} & 0 & 0 & 0 & 0 & 0 & 0 & 0\\ \frac{\delta }{G_{0w}} & -(\mu_{l}+\psi ) & 0 & 0 & 0 & 0 & 0 & 0\\ 0 & b_{f}\psi & -\mu_{fu} & 0 & 0 & 0 & 0 & 0\\ 0 & b_{m}\psi & 0 & -\mu_{mu} & 0 & 0 & 0 & 0\\ 0 & 0 & 0 & 0 & -\delta -\mu_{ew} & 0 & \phi_{w} & 0\\ 0 & -\frac{G_{0w}}{\delta }\left(\psi +\mu_{l}\right) & 0 & 0 & \frac{\delta }{G_{0w}} & -G_{0w}\left(\mu_{l}+\psi \right) & 0 & 0\\ 0 & 0 & 0 & 0 & 0 & b_{f}\psi & -\mu_{fw} & 0\\ 0 & 0 & 0 & 0 & 0 & b_{m}\psi & 0 & -\mu_{mw} \end{array}\right)\;.$$

By applying the same argument we used in the previous proof, we consider the 4×4 diagonal elements:

$$A_{\mathrm{CIE}}=\left(\begin{array}{cccc} -\delta -\mu_{eu} & 0 & 0 & 0\\ \frac{\delta }{G_{0u}} & -(\mu_{l}+\psi ) & 0 & 0\\ 0 & b_{f}\psi & -\mu_{fu} & 0\\ 0 & b_{m}\psi & 0 & -\mu_{mu} \end{array}\right)$$

and

$$D_{\mathrm{CIE}}=\left(\begin{array}{cccc} -\delta -\mu_{ew} & 0 & \phi_{w} & 0\\ \frac{\delta }{G_{0w}} & -G_{0w}\left(\mu_{l}+\psi \right) & 0 & 0\\ 0 & b_{f}\psi & -\mu_{fw} & 0\\ 0 & b_{m}\psi & 0 & -\mu_{mw} \end{array}\right)\;.$$

Observe that $A_{CIE}$ is a Metzler matrix (negative diagonal elements) and it is lower triangular, which means that its eigenvalues are the same negative diagonal elements. Thus, $A_{CIE}$ is Metzler stable. Now, we need to analyze the stability conditions of $D_{CIE}$. For this purpose, consider the 3×3 leading submatrix of $D_{CIE}$:

$$D_{s2}=\left(\begin{array}{ccc} -\delta -\mu_{ew} & 0 & \phi_{w}\\ \delta /G_{0w} & -G_{0w}\left(\mu_{l}+\psi \right) & 0\\ 0 & b_{f}\psi & -\mu_{fw} \end{array}\right)=\left(\begin{array}{cc} A_{3} & B_{3}\\ C_{3} & D_{3} \end{array}\right)\;.$$

By applying a similar argument as before, $A_{3}$ is Metzler stable, and the number $D_{3}-C_{2}A_{2}^{-1}B_{2}$ satisfies the inequality:

(A6) $$D_{3}-C_{3}A_{3}^{-1}B_{3}=\mu_{fw}\left(\frac{1}{G_{0w}}-1\right)<\;0,\;\;\mathrm{if}\;\;G_{0w}>1\;.$$

Hence, when $G_{0w}>1$, $D_{s2}$ is Metzler stable, so is the matrix $D_{CIE}$. Therefore, we conclude that Equation (A6) ensures the stability of $J_{CIE}$. □

## Appendix C. Seasonality Fitting

We extracted CHIRPS monthly rainfall data during 2016–2020 for the department of Grand Anse [31] in Haiti using R Studio version 4.2.1. The median values for each month over these five years were calculated in Table A1. Most rainfall occurs during two rainy seasons in Grand Anse, April–June and September–November, and the dry season occurs in December–March and July–August.

We employed a smoothing spline with a periodicity of 12 months and a smoothing parameter of 0.9 (implemented in MATLAB) to fit the data points. Assuming that the carrying capacity level was proportional to the amount of rainfall, we rescaled the data points by a constant coefficient (≈1400) so that the obtained spline predicted an annual average carrying capacity of around $K_{l}=2\times 10^{5}$. This was the baseline level assumed in the main text (Table 3). The fitted curve and the rescaled data points are shown in Figure A1.

We also extracted the aridity index data in the region, where only the averaged data for the years 1970–2000 were readily available (Table A1). The global aridity index is a function of the evapotranspiration processes and rainfall deficit for potential vegetative growth. It is calculated as the mean annual precipitation mean divided by annual reference evapotranspiration. We employed a similar smoothing spline method as described above, and the fitted curve (dashed line in Figure A1) had a similar trend as the rainfall data. The mean aridity index over the year was 1.13 with 79% of the time classified as humid (index above 0.65). Thus, to approximate the carrying capacity, we followed the curve rescaled from the rainfall data.

The temperature variance in the region is mild (see Table A1 row 3). Thus, we assumed temperature independence in our model parametrization.

**Table A1:** Seasonality data for the department of Grand Anse in Haiti. Row 1: monthly median rainfall (in mm) from 2016–2020. Row 2: mean aridity index for years 1970–2000. Row 3: mean monthly land surface temperature for 2018–2020 (in Celsius).

	Jan	Feb	Mar	Apr	May	Jun	Jul	Aug	Sep	Oct	Nov	Dec
Rainfall	59	75	82	131	302	110	94	107	205	178	216	62
Aridity	0.6	0.5	0.5	0.9	1.8	1.2	0.8	1.2	1.1	2.3	1.4	0.8
Temp.	25.7	27.6	28.9	29.8	29.8	29.6	29.2	29.7	29.3	27.8	26.4	25.7
